# Nursing workload in intensive care units estonia based on nursing activities score

**DOI:** 10.1186/2197-425X-3-S1-A924

**Published:** 2015-10-01

**Authors:** E Keldo, V Toome

**Affiliations:** North Estonia Medical Centre, Postoperative ICU, Tallinn, Estonia; North Estonia Medical Cantre, Tallinn, Estonia

## Introduction

High nursing workload variability in intensive care units makes the planning of nursing manpower problematic. Nevertheless measuring nursing workload is essential for the internest of nurses, the safety of patients and the efficience of using the financial resources. Thereby its important to measure it using valid and reliable tool. Many authors has described Nursing Activities Score as the best available tool for measuring the nursing workload in intensive care units.

## Objectives

To describe the nursing workload and to assess an optimum of nursing manpower and nurse - patient ratio in third degree adult intensive care units of regional and central hospitals in Estonia.

## Methods

Quantitative, descriptive, prospective reseach. The data was collected with Nursing Activities Score between 03.11.2014 - 30.11.2014. The NAS was registered in the day and in the night shift per patient. The mean NAS/per nurse/per shift, NAS/per patient/per shift were calculated and compared between the day and night shifts and between the weekdays and in the weekends and is based on 6 ICU from 3 hospitals. Also the optimum of nursing manpower and the nurse - patient ratio were assessed.

## Results

The Sample consisted of 2806 NAS records from 10 ICU-s in Estonia. The mean NAS/Nurse in day shifts was 85,3% and NAS/Patient was 65,14% which correlates well with ohter studies. The workload has a great variety compared with different shifts and units (NAS/per nurse/ per shift =17,52-148,74%. ) In addition the differences between the day and night shifts and the real and optimal nursing manpower based on NAS were discovered.

## Conclusions

The nurses in adult intensive care units in Estonian hospitals are most of the time occupied with nursing activities and ICU patients need different amount of the nursing activities. The nursing manpower in our ICU-s had too little variety and the official nurse - patient ratio 1 : 2 is inadequate from the perspective of patient safety and nurses wellbeing. In the units that were attended on the survey, the mean optimal nurse - patient ratio was 1 : 1,5. Based on the fact that the NAS results are comperable with other studies from different countries it is possible to claim that Nursing Activities Score is a reliable tool also for Estonian ICU-s for measuring the nursing workload.

## References

[1] Debergh DP, Myny D, Van Herzeele I, Van Maele G, Miranda DR, Colardyn F (2012). Measuring the nursing workload per shift in the ICU. Intensive Care Medicine.

[2] Miranda DR, Nap R, de Rijk A, Schaufeli W, Iapichino G, and the members of the TISS Working Group (2003). Nursing activities score. Critical Care Medicine.

